# Effect of different anaesthetic techniques on gene expression profiles in patients who underwent hip arthroplasty

**DOI:** 10.1371/journal.pone.0219113

**Published:** 2019-07-25

**Authors:** Renata Alleva, Andrea Tognù, Marco Tomasetti, Maria Serena Benassi, Laura Pazzaglia, Hanna van Oven, Ettore Viganò, Nicola De Simone, Ilaria Pacini, Sandra Giannone, Sanjin Gagic, Raffaele Borghi, Sara Picone, Battista Borghi

**Affiliations:** 1 Department of Biomedical and Neuromotor Sciences, University of Bologna, Bologna, Italy; 2 Department of Anaesthesia and Postoperative Intensive Care, Rizzoli Orthopaedic Institute, Bologna, Italy; 3 Department of Clinical and Molecular Sciences, Polytechnic University of Marche, Ancona, Italy; 4 Laboratory of Experimental Oncology, Rizzoli Orthopaedic Institute, Bologna, Italy; 5 Research Unit of Anaesthesia and Pain Therapy, Rizzoli Orthopaedic Institute, Bologna, Italy; Public Library of Science, UNITED KINGDOM

## Abstract

**Objectives:**

To investigate the modulation of genes whose expression level is indicative of stress and toxicity following exposure to three anaesthesia techniques, general anaesthesia (GA), regional anaesthesia (RA), or integrated anaesthesia (IA).

**Methods:**

Patients scheduled for hip arthroplasty receiving GA, RA and IA were enrolled at Rizzoli Orthopaedic Institute of Bologna, Italy and the expression of genes involved in toxicology were evaluated in peripheral blood mononuclear cells (PBMCs) collected before (T0), immediately after surgery (T1), and on the third day (T2) after surgery in association with biochemical parameters.

**Results:**

All three anaesthesia methods proved safe and reliable in terms of pain relief and patient recovery. Gene ontology analysis revealed that GA and mainly IA were associated with deregulation of DNA repair system and stress-responsive genes, which was observed even after 3-days from anaesthesia. Conversely, RA was not associated with substantial changes in gene expression.

**Conclusions:**

Based on the gene expression analysis, RA technique showed the smallest toxicological effect in hip arthroplasty.

**Trial registration:**

*ClinicalTrials*.*gov* number NCT03585647.

## Introduction

Arthroplasty surgery is nowadays the technique of choice to treat different articular conditions, such as knee or hip arthritis. In hip arthroplasty, regional anaesthesia (RA) represents one of the preferred options for anaesthesia, due to its safety and convenience for both patients and surgeons [1]. Peripheral nerve block is frequently used, reducing the dose and thus limiting the adverse effects of opioid and non-opioid analgesics [2]. Use of RA is known to prevent or attenuate excessive stress response during and after surgery. RA markedly decreased recovery time and postoperative pain, with similar levels of patient satisfaction when compared to GA [3,4].

Besides, multi-modal analgesia is increasingly used to reduce postoperative pain and accelerate rehabilitation.^3^ Integrated anaesthesia (IA), which consists of lumbar plexus block plus spinal anaesthesia, integrated with GA, represents another option, and has been shown to reduce intra-operative anaesthetic drugs requirement and to provide better post-operative pain relief in shoulder arthroscopic surgeries [5]. As of today, the cytotoxic effects of different anaesthesia techniques have not been clearly elucidated. A few studies have evaluated the effect of anaesthesia on gene expression (transcriptome) in cells. Lowes et al [6], reported that isoflurane anaesthesia affects differential gene expression in the brain of anesthetized rats compared to control. Isoflurane alters several genes involved with neurotransmitter transport, signalling and cellular structure [7], as well as genes involved in drug metabolism and clock gene expression [8].

In this study, we analysed differentially regulated genes involved in oxidative stress and toxicology in peripheral blood mononuclear cells (PBMCs) of patients who underwent arthroplasty under three different anaesthetic methods. We hypothesized that anaesthesia procedures trigger toxicity, thus inducing changes in the mRNA profile. The results may provide a more profound understanding of the molecular mechanism of anaesthesia and in overcoming the adverse effects arising from their use.

## Methods

### Ethics statement

The study was carried out according to the Helsinki Declaration and written informed consent was obtained from all participants. Ethical approval for this study (Ethical Committee N° 12630) was provided by the Regional Ethical Committee of the Rizzoli Orthopaedic Institute, via pupilli 1–40136, Bologna, Italy, (Chairperson Prof. Gian Paolo Salvioli) on 12 June 2014 (S1 File).

### Study population

**Fig 1:**
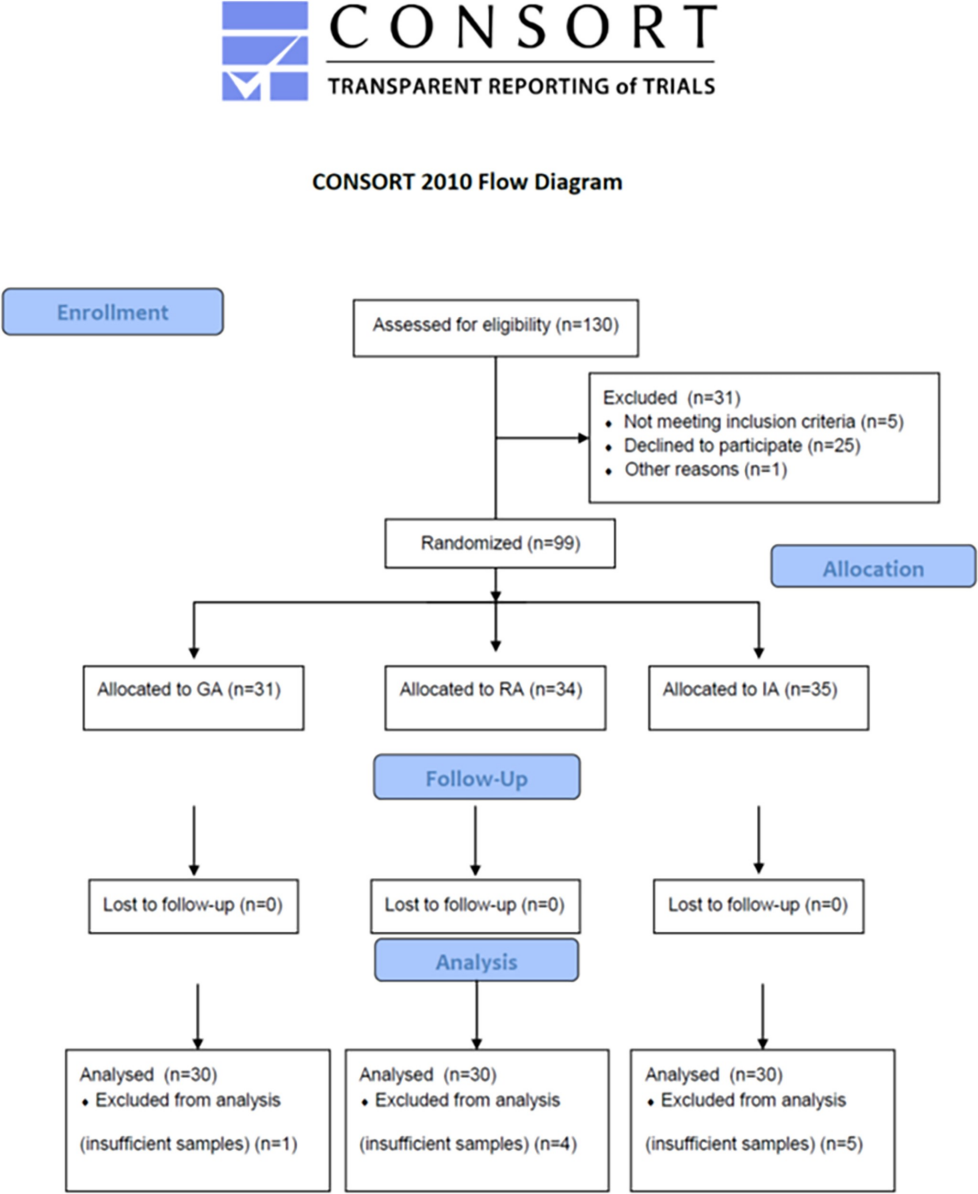
Overview of study design according to CONSORT format. Accordantly to eligibility, 130 patients were enrolled. Of them, 31 patients were excluded from the study, then 99 patients were randomised in the three anaesthesia groups.

Patients undergoing elective hip arthroplasty were recruited and followed-up from September 2014 to November 2016 at the ‘Rizzoli’ Orthopaedic Institute, Bologna, Italy. The study design is shown in Fig 1, according to CONSORT Checklist (S2 File). The sample size was estimated according to Lee-Whitmore and G-Power F-test for ANOVA fixed effects, omnibus, one-way [9]. Taking in account a power of 0.8 and a Bonferroni correction with a significance of 0.05, the minimum sample size was established for 30 patients of each group. By considering a 10% of drop-out, the chosen sample size was 33 patients per group which leads to a total sample size of 99 patients.

By using a computer-generated randomization table, patients were randomly consecutively allocated to receive general (GA group, n = 30), regional (RA group, n = 30), or integrated (IA group, n = 30) anaesthesia. After arrival in the operating theatre, an 18-gauge intravenous cannula was placed at the forearm, and then all patients were pre-medicated with 0.05 mg/kg intravenous (IV) midazolam. A 5-mL/kg/hour intravenous infusion with ringer lactate was then started without prophylactic volume expansion. The duration of the procedure was 110 ± 47 min. in group GA, 108 ± 31 min. in group RA, and 107 ± 41 min. in group IA (p = 0.68). Patients with contraindication to spinal anaesthesia or lumbar catheter placement, as well as obese patients, with arterial hypertension not controlled by oral medication, severe pulmonary, cardiovascular, renal, hepatic, cerebrovascular, or psychiatric diseases were excluded from the study. Accounting for a 30% drop-out rate, a total of 130 patients were enrolled. The study protocol (S3 File) was registered at www.clinicaltrials.gov (NCT03585647) after enrolment of participants as the study later was defined as a Clinical Trial and all related clinical trials have been registered. All participants provided written informed consent based on documents approved by Institute Institutional Review Board.

### Anaesthesia procedures

GA was induced by intravenous fentanyl (1 mcg/kg) and propofol (2 mg/kg), followed by vecuronium bromide (0.1 mg/kg) to facilitate tracheal intubation, then GA was maintained using a 50% air/oxygen mixture and sevoflurane. The end-tidal concentration of sevoflurane was adjusted to maintain heart rate and blood pressure values within 20% of baseline. Mechanical ventilation was regulated to maintain the end-tidal CO_2_ partial pressure ranging between 4.3 and 5.1 Kilopascal (kPa).

RA: included continuous lumbar plexus block, performed by or under supervision of an experienced operator using a nerve stimulator (Stimuplex-HNS 11, B. Braun Melsungen, Germany) and Braun Contiplex-Tuohy Continued Peripheral Nerve Block Set.

The landmark used to locate the needle insertion point was the soft tissue depression at the iliac crest prominence, the most anterior and cranial aspect of the crest. A total dose of 20 ml of 0.5% Levobupivacaine was administered at the time of catheter placement. Then, dural puncture was performed at the L3-L4 interspace using a 25-Gauge Whitacre spinal needle (Becton-Dickinson, New Jersey, USA) with the midline approach using 3 ml of 0.5% Levobupivacaine.

IA: First, the patients received regional anaesthesia (lumbar plexus block + spinal anaesthesia) as described above. Then GA was induced by propofol 1% and a laryngeal mask airway of appropriate size was inserted. GA and mechanical ventilation were maintained as described above.

Postoperative analgesia: patients in the RA and IA groups received a continuous lumbar plexus infusion of levobupivacaine 0.25% at 7 mL/h injected through an elastomeric pump, whereas the GA group was treated with a continuous infusion of 1 mg/h morphine after an intravenous bolus of 0.1 mg/kg. If such analgesic treatment was not sufficient to relieve the postoperative pain, a rescue dose of 30 mg ketorolac (Recordati, Italy) was available for the patients. The pain intensity level was evaluated by Numerical Rating Scale (NRS) values. The prediction of the operative risk has been evaluated by American Society of Anaesthesiologists (ASA) physical status classification [10].

### Blood samples

Whole blood samples (10 mL) were obtained from all enrolled patients at three time points: early morning on the operation day (T0), immediately after surgery (T1) and third day (T2) after surgery. The samples were collected in heparin tubes and PBMCs were isolated as previously described for gene expression evaluation [11]. Briefly, blood samples (6 ml) were layered onto 4 ml of Lympholyte-H (Cederlane, Hornby, Ontario, Canada) and centrifuged at 1000 g (20°C, 30 min). After centrifugation, the cloudy layer was collected and placed in a 15 ml Falcon tube, filled with PBS, pH 7.4, and centrifuged at 1000 g (20°C, 15 min). After removing the supernatant, the pellet of PBMCs containing 80–90% of lymphocytes was collected and stored at -80°C for RNA extraction. Whole blood and serum obtained after centrifugation were used for the analysis haematological and biochemical, such as Glutamate Oxaloacetate Transaminase (GOT), Glutamate-Pyruvate Transaminase (GPT), Bilirubin (BIL), Creatinine (CREA), Creatine Phosphokinase (CPK), and Haemoglobin (HB).

### Microarray analysis

Total RNA was extracted from PBMCs using PerfectPure RNA Kit (5Prime, Hamburg, Germany) according to the manufacturer’s instructions. The cDNA was synthesized using RT^2^-First Strand Kit (SA Biosciences, Frederick, MD, USA) according to the manufacturer’s instructions. Human Stress and Toxicity PathwayFinder RT^2^ Profiler PCR Expression Array (PAHS-003 SABiosciences) was used for gene expression profiling. The expression of 84 stress- and toxicity-related genes was assessed by qRT- PCR (Mastercycler EP Realplex, Eppendorf, Milano, Italy) using RT^2^ SYBR Green qPCR Master Mix (SABiosciences). A set of controls (6 housekeeping genes, 3 control genes for quality of the retro-transcription and 3 control genes for qPCR) were included in the array. Among the tested housekeeping genes, *GAPDH* showed smaller changes in its expression across different sample groups (differences in Ct values < 1). Gene-specific products were then normalized to *GAPDH* and expressed as fold change (2^-ΔΔCt^). The PCR array raw dataset are shown in theS4 File.

### Gene ontology analysis

Gene ontology analysis with Benjamini and Adchberg false discovery rate (FDR) correction, (cut-off<0.05), was performed to identify the sets of genes grouped to biological process and molecular function (nodes) that are significantly different as expressed by the anaesthetic procedures. Of all genes being significantly expressed, the fold change versus preoperative control was determined. The data were analysed by Cytoscape software.

### Quantitative RT-PCR analysis

Total RNA from PBMCs was obtained using the RNeasy Mini Kit (Qiagen), according to the manufacturer's instructions. The concentration of RNA was determined spectrophotometrically at 260 nm (NanoDrop ND-1000, Thermo Scientific, Wilmington, USA). The cDNA was synthesized using the High-Capacity cDNA Reverse Transcription Kit (Applied Biosystems). The expression of selected genes was quantified using the TaqMan system (Applied Biosystems, Foster City, CA, USA), using the Mastercycler EP Realplex instruments (Eppendorf). The *GAPDH* was used for normalization and the results were expressed as ΔCT, and fold changes in relative mRNA expression were calculated using the equation 2^-ΔΔCT^.

### Statistical analysis

Results were expressed as mean ± S.D. Normalization and microarray analysis were performed by RT^2^ Profiler PCR Array Analysis software version 3.5 (SABiosciences), and two-tailed Student’s t-test was used to compare gene expression at T0 versus T1 and T2 time points. Comparisons among groups of data (age and BMI) were made using one-way analysis of variance (ANOVA) with Tukey post-hoc analysis. The Chi-square test was used to compare categorical variables (gender and ASA). ANOVA repeated measure with the Sidak post-hoc test was used to evaluate the changes of biochemical parameters (GOT, GPT, BIL, CREA, CPK, HB) in each group over time (T0, T1, T2). Generalized linear model (GLM) multivariate regression analysis was used to model the changes (ΔT = T0-T2) of biochemical parameters (GOT, GPT, BIL, CREA, CPK, HB) using continuous variables (age and body mass index, BMI) as covariates, and categorical variables (anaesthetic methods, gender and ASA) as fixed factors. The data were analysed by the Statistical Package Social Sciences (version 19) software (SPSS, Chicago, IL, USA) and p-values less than 0.05 were considered significant.

## Results

There were no significant differences in age, BMI, gender, clinic-pathologic parameters and ASA scores among GA, RA and IA groups. However, an increase of transaminases (GOT and GPT) was observed on the third postoperative day in patients underwent to GA as result of a hepatic damage (Table 1).

**Table 1:** Demographic characteristics and biochemical parameters of the enrolled subjects.

		**GA (n = 30)**	**RA (n = 30)**	**IA (n = 30)**
Age (years)		58.1±12.1	62.1±9.4	63.1±11.8
BMI (Kg/m^2^)		26.6±2.6	27.6±3.0	26.7±3.8
Gender (M/F %)		46/54	44/56	39/61
ASA class (I/II %)		46/54	38/62	25/75
**Biochemical parameters**	**Time-points**	**GA (n = 30)**	**RA (n = 30)**	**IA (n = 30)**
GOT (mg/dl)	T-0	18.6±4.7	25.6±17.0	18.8±2.8
	T-1	23.8±8.5	27.7±9.8	27.1±8.9
	T-2	42.9±36.5*	31.1±18.2	25.0±7.8
GPT (mg/dl)	T-0	17.8±8.4	26.6±18.1	17.3±8.4
	T-1	15.9±7.8	19.6±8.8	15.4±6.3
	T-2	36.9±37.5*	23.9±17.7	14.7±6.0
BIL (mg/dl)	T-0	1.16±0.80	0.74±0.50	0.72±0.34
	T-1	1.24±0.92	1.15±0.65	1.26±0.64
	T-2	0.78±0.48	0.62±0.23	0.69±0.25
CREA (mg/dl)	T-0	0.85±0.18	0.83±0.19	0.83±0.14
	T-1	0.79±0.17	0.79±0.15	0.80±0.11
	T-2	0.76±0.16	0.76±0.15	0.76±0.12
CPK (mg/dl)	T-0	217±317	133±124	120±83
	T-1	647±420	551±387	554±276
	T-2	468±293	472±500	488±280
HB (mg/dl)	T-0	11.7±0.9	10.5±4.4	11.6±4.2
	T-1	10.5±1.0	11.2±1.7	11.2±1.7
	T-2	9.4±1.4	9.9±0.9	10.7±1.3

General anaesthesia, GA; Regional anaesthesia, RA; Integrated anaesthesia, IA: Body mass index, BMI; Glutamate Oxaloacetate Transaminase, GOT; Glutamate-Pyruvate Transaminase, GPT; Bilirubin, BIL; Creatinine, CREA; Creatine phosphokinase, CPK; Hemoglobin, HB.

* T0 vs T1 and T2 time points, with p<0.05.

**Fig 2:**
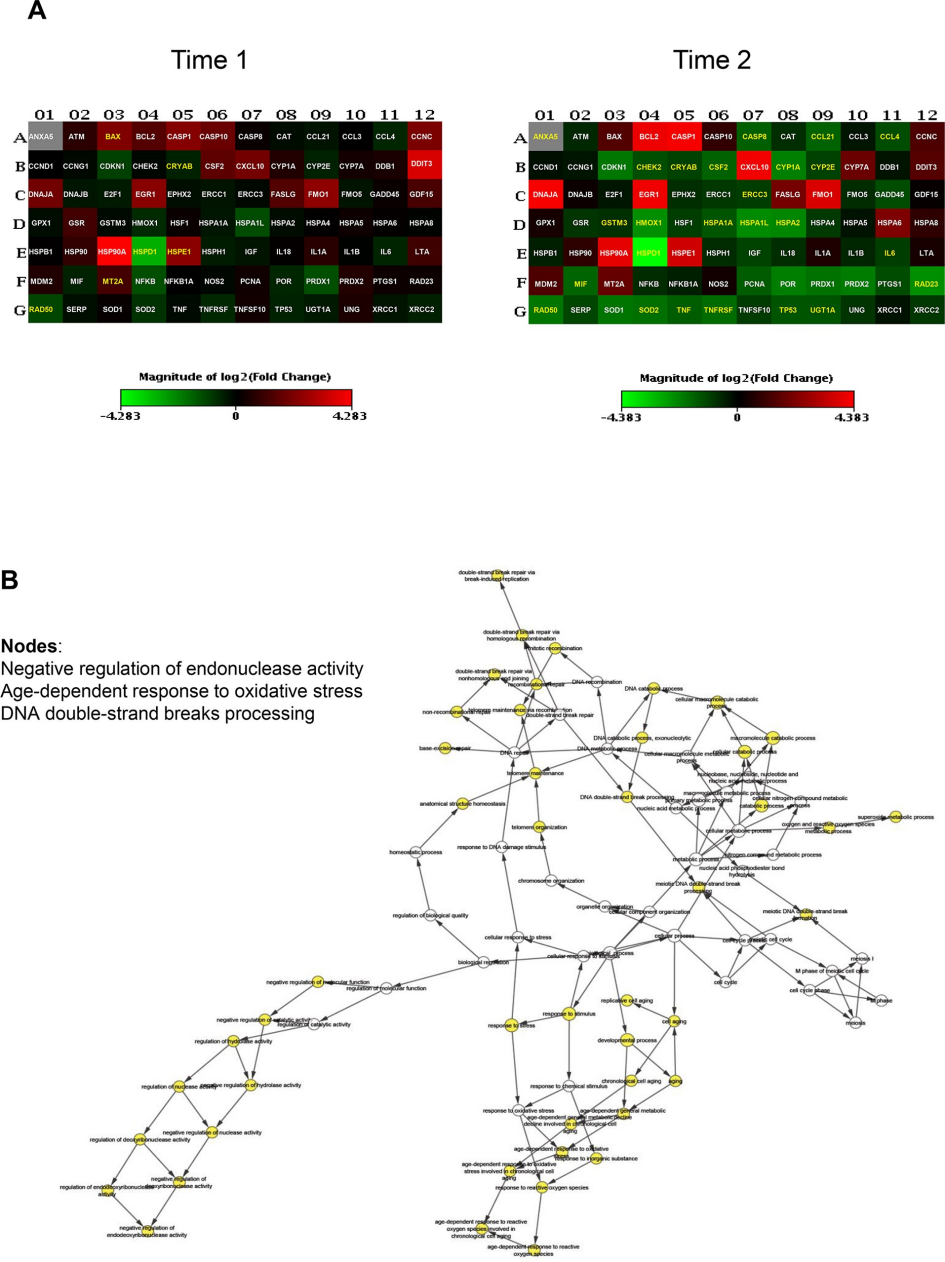
Gene expression analysis in patients receiving general anaesthesia (GA). (A) Heatmap of significant gene expression in PBMCs of patients (n = 3) undergoing elective hip arthroplasty receiving GA, immediately after operation (T1) and on the third postoperative day (T2), adjusted at p-value less than 0.05. Genes with greater and lower abundance after anaesthesia (FC, fold change) are shown in red and green, respectively, with significance highlighted in yellow. Normalization and microarray analysis were performed by RT^2^ Profiler PCR Array Analysis software version 3.5 (SABiosciences). (B) Gene-gene interaction analysis of the significantly differentially expressed genes showing the networks of deregulated pathways at T2 time point.

**Fig 3:**
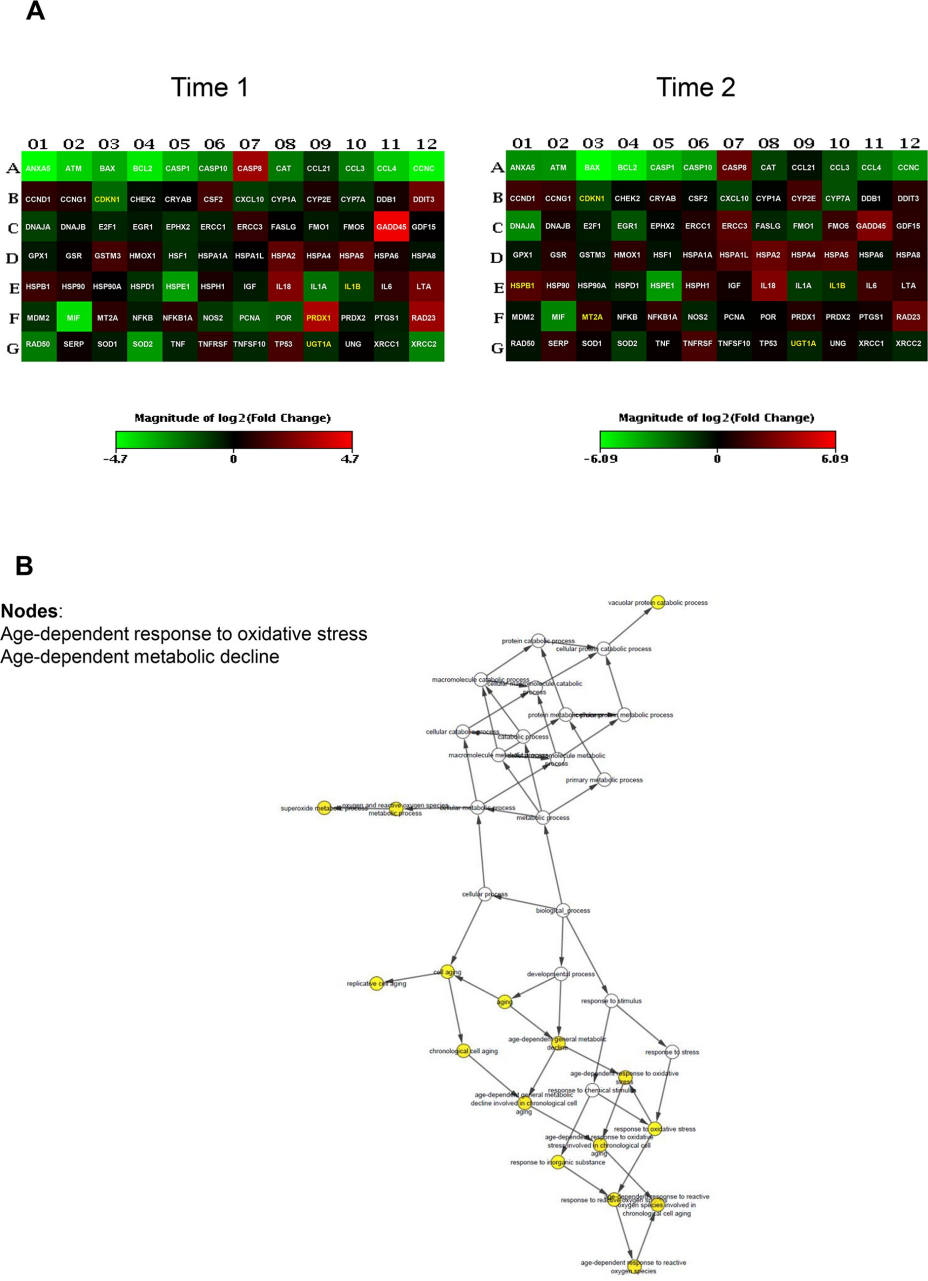
Gene expression analysis in patients receiving regional anaesthesia (RA). (A) Heatmap of significant gene expression in PBMCs of patients (n = 3) undergoing elective hip arthroplasty receiving RA, immediately after operation (T1) and on the third postoperative day (T2), adjusted at p-value less than 0.05. Genes with greater and lower abundance after anaesthesia (FC, fold change) are shown in red and green, respectively, with significance highlighted in yellow. Normalization and microarray analysis were performed by RT^2^ Profiler PCR Array Analysis software version 3.5 (SABiosciences). (B) Gene-gene interaction analysis of the significantly differentially expressed genes showing the networks of deregulated pathways at T2 time point.

**Fig 4:**
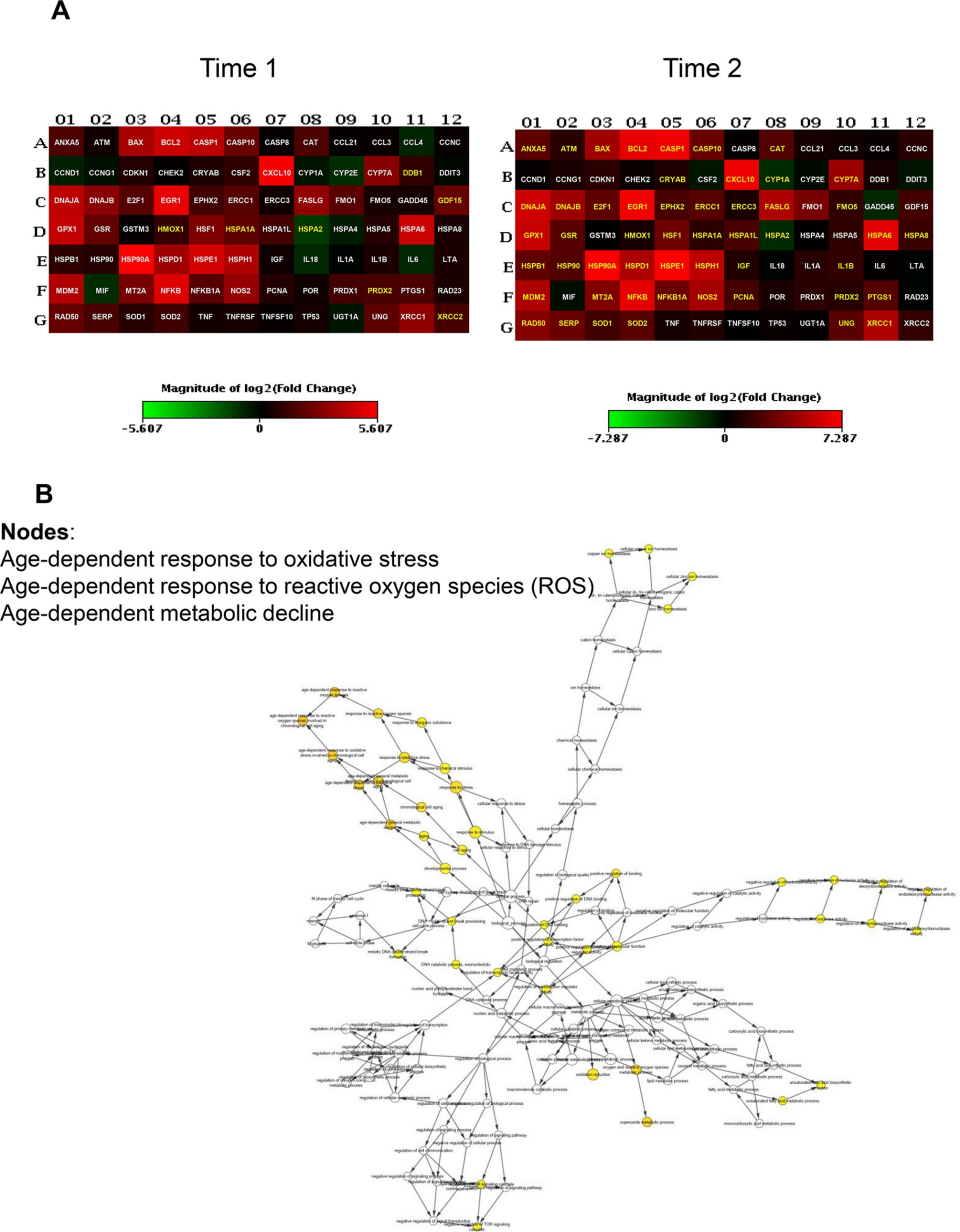
Gene expression analysis in patients receiving integrated anaesthesia (IA). (A) Heatmap of significant gene expression in PBMCs of patients (n = 3) undergoing elective hip arthroplasty receiving IA, immediately after operation (T1) and on the third postoperative day (T2), adjusted at p-value less than 0.05. Genes with greater and lower abundance after anaesthesia (FC, fold change) are shown in red and green, respectively, with significance highlighted in yellow. Normalization and microarray analysis were performed by RT^2^ Profiler PCR Array Analysis software version 3.5 (SABiosciences). (B) Gene-gene interaction analysis of the significantly differentially expressed genes showing the networks of deregulated pathways at T2 time point.

**Fig 5:**
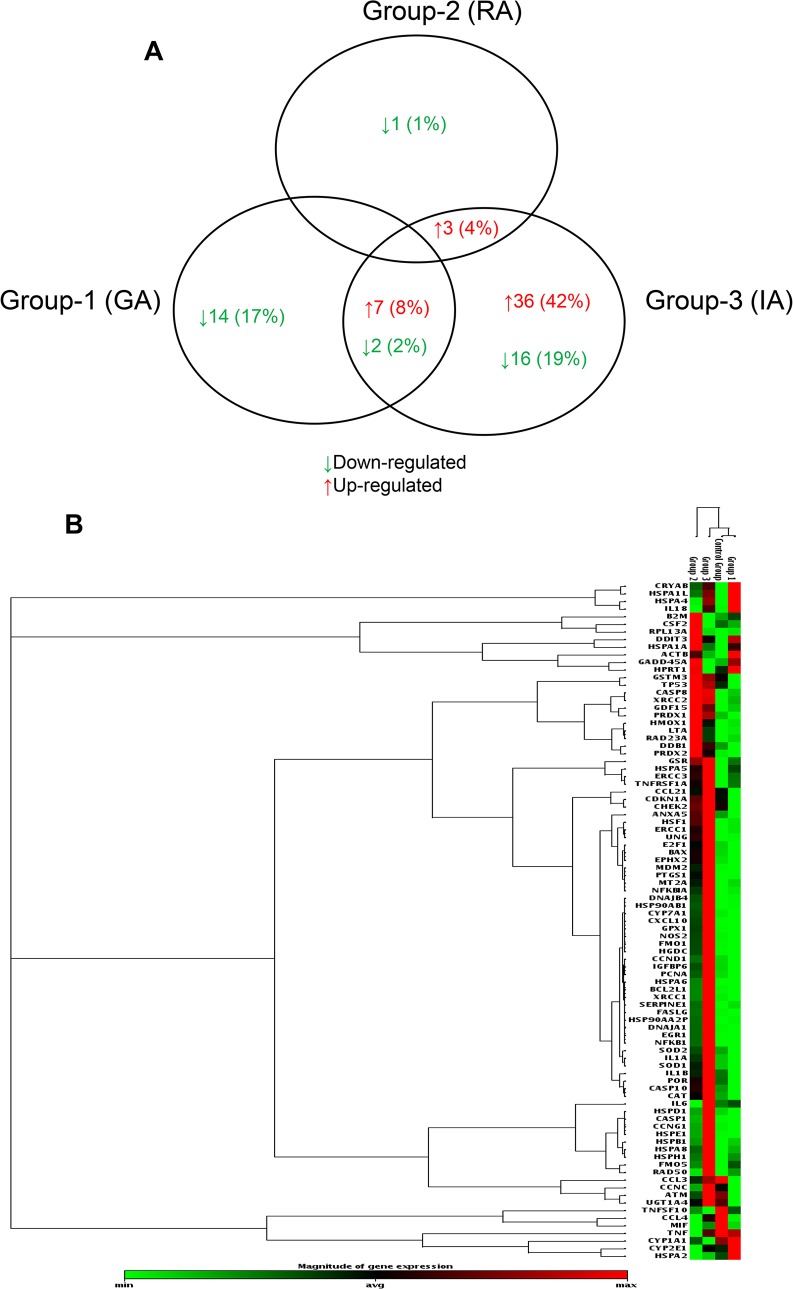
Venn diagram and gene clustering. (A) Venn diagram that shows differentially expressed genes that were shared among three anaesthesia methods. Cluster analysis of differentially expressed genes in general anaesthesia (Group-1), regional anaesthesia (Group-2), and integrated anaesthesia (Group-3) at third postoperative day (T2), respect to control Group (before anaesthesia, T0). (B) Gene clustering by their expression levels.

To uncover differentially expressed genes in relation to anaesthesia procedures, we performed a screening phase where 84 genes involved in cell stress and toxicity were evaluated in PBMCs from a sub-population of 9 patients. Patients undergoing to arthroplasty surgery under GA (n = 3, age = 61±6; M/F = 1/2; BMI = 27±3, ASAI/II = 0/3), receiving RA (n = 3, age = 63±5; M/F = 2/1; BMI = 29±1, ASAI/II = 1/2), or receiving IA (n = 3, age = 64±1; M/F = 1/2; BMI = 29±4, ASAI/II = 0/3), before (T0) and after operation (T1) and on postoperative day three (T2) were randomly selected for gene expression profiling. The complete list of genes used to identify deregulated pathways is provided on S1 Table. Differentially expressed genes within different anaesthesia techniques at T1 and T2 time points respect to T0 were identified as having a ± 2-fold expression change and a p-value less than 0.05. As shown in Fig 2A, GA modulates stress-responsive genes such as *RAD50*, a protein involved in DNA double-strand break repair, and the small heat shock protein (HSP) family. Most genes were down regulated; the fold changes of the deregulated genes at time points T1 and T2 are reported in Table 2. Gene ontology analysis (biological and molecular process) revealed significantly altered genes involved in age-dependent response to reactive oxygen species (ROS) and in the negative regulation of nuclease and endonuclease activity by affecting DNA binding (Fig 2B). Few genes were induced in the RA group, mostly involved in the age-dependent response to metabolic and oxidative stress. This is represented graphically in Fig 3A and 3B and fold changes showed in Table 3, where most of the genes were under-expressed or not affected by the anaesthesia. Conversely, induction of gene expression was observed in the IA group. The gene expression profile drastically changes at T2 time point with the 42% of up-regulated genes and 19% of down-regulated genes (Table 4). Gene ontology analysis identified nodes involved in the age-dependent response to metabolic decline and oxidative stress (Fig 4A and 4B). Fig 5A and 5B shows the Venn diagram of the distribution of the co-deregulated genes across the anaesthesia groups and clustering analysis at T2 time point.

**Table 2:** Deregulated gene expression in PBMCs of patients (n = 5) undergoing elective hip arthroplasty receiving general anaesthesia (GA), immediately after operation (T1) and on the third postoperative day (T2).

Genes	T1		T2	
	FC	p-value	FC	p-value
*ANXA5*	2.5	0.153	**4.4**	**0.010**
*BAX*	**4.4**	**0.048**	2.6	0.384
*CASP8*	1.1	0.508	**-3.1**	**0.017**
*CCL21*	-1.3	0.551	**-3.2**	**0.029**
*CCL4*	-1.6	0.276	**-2.5**	**0.044**
*CHEK2*	-1.1	0.653	**-2.5**	**0.013**
*CRYAB*	**-1.5**	**0.039**	**-2.1**	**0.006**
*CSF2*	3.0	0.219	**-3.2**	**0.023**
*CYP1A1*	1.4	0.253	**-4.6**	**0.009**
*CYP2E1*	-1.4	0.065	**-2.2**	**0.018**
*EGR1*	5.0	0.166	18.0	0.354
*ERCC3*	1.1	0.444	**-2.1**	**0.001**
*FASL*	2.5	0.186	4.1	0.377
*GSTM3*	-1.1	0.701	**-1.8**	**0.011**
*HMOX1*	-1.6	0.285	**-5.5**	**0.008**
*HSPA1A*	-1.3	0.298	**-2.8**	**0.027**
*HSPA1L*	-2.4	0.092	**-5.3**	**0.022**
*HSPA2*	-1.1	0.343	**-5.1**	**0.007**
*HSP90AB1*	19.4	0.059	15.8	0.333
*HSPD1*	**-7.7**	**0.005**	**-20.9**	**0.009**
*HSPE1*	**5.1**	**0.010**	12.2	0.356
*IL6*	-1.5	0.080	**-2.1**	**0.043**
*MIF*	-1.5	0.451	**-3.3**	**0.021**
*MT2A*	2.2	0.234	3.3	0.055
*RAD23A*	1.1	0.710	**-5.5**	**0.033**
*RAD50*	**-2.2**	**0.002**	**-4.1**	**0.006**
*SOD2*	-1,8	0.177	**-3.8**	**0.047**
*TNF*	-1.1	0.850	**-3.0**	**0.015**
*TNFRSF1A*	-1.6	0.391	**-3.6**	**0.042**
*TNFSF10*	1.2	0.498	-2.1	0.085
*TP53*	-1.5	0.141	**-2.8**	**0.002**
*UGT1A4*	-1.5	0.137	**-2.7**	**0.002**

The genes were expressed as Fold-change (FC) respect to time (T0). The significant down- and up-regulated genes are highlighted in bold.

**Table 3:** Deregulated gene expression in PBMCs of patients (n = 5) undergoing elective hip arthroplasty receiving regional anaesthesia (RA), immediately after operation (T1) and on the third postoperative day (T2).

Genes	T1		T2	
	FC	p-value	FC	p-value
*CDKN1A*	**-3.8**	**0.029**	**-2.9**	**0.015**
*GADD45A*	22.2	0.06	8.5	0.530
*HSPB1*	2.2	0.107	**3.9**	**0.026**
*IL1B*	**-2.2**	**0.022**	**-2.1**	**0.025**
*MT2A*	1.8	0.093	**2.0**	**0.043**
*PRDX1*	**9.2**	**0.047**	1.8	0.783
*SOD2*	-7.2	0.060	-2.4	0.140
*UGT1A4*	**-1.8**	**0.032**	**-1.8**	**0.034**

The genes were expressed as Fold-change (FC) respect to time (T0). The significant down- and up-regulated genes are highlighted in bold.

**Table 4:** Deregulated gene expression in PBMCs of patients (n = 5) undergoing elective hip arthroplasty receiving integrated anaesthesia (IA), immediately after operation (T1) and on the third postoperative day (T2).

Genes	T1		T2		Genes	T1		T2	
	FC	p-value	FC	p-value		FC	p-value	FC	p-value
*ANXA5*	3.3	0.270	**6.2**	**0.005**	*HSPA5*	1.8	0.228	2.4	0.060
*ATM*	1.3	0.426	**2.3**	**0.003**	*HSPA6*	22.2	0.176	**90.0**	**0.004**
*BAX*	13.5	0.090	**24.7**	**0.001**	*HSPA8*	1.5	0.339	**3.1**	**0.008**
*BCL2L1*	23.4	0.199	**99.3**	**0.0003**	*HSPB1*	2.0	0.292	**6.9**	**0.006**
*CASP1*	18.0	0.173	**123.1**	**0.0001**	*HSP90AA2P*	1.8	0.404	**4.9**	**0.002**
*CASP10*	5.1	0.104	**8.26**	**0.0002**	*HSP90AB1*	48.7	0.158	**147.0**	**0.0003**
*CAT*	3.2	0.127	**5.1**	**0.032**	*HSPD1*	11.6	0.123	**32.7**	**0.0004**
*CCNC*	1.1	0.902	**2.3**	**0.066**	*HSPE1*	26.1	0.164	**156.1**	**0.0005**
*CRYAB*	1.6	0.199	**1.8**	**0.013**	*HSPH1*	14.5	0.159	**42.7**	**0.0008**
*CXCL10*	44.3	0.126	**120.3**	**0.001**	*IGFBP6*	1.2	0.467	**1.9**	**0.018**
*CYP1A1*	-1.3	0.479	**-2,3**	**0.030**	*IL18*	-1.8	0.161	-1.0	0.875
*CYP7A1*	8.1	0.170	**22.3**	**0.0003**	*IL1A*	-1.1	0.376	2.2	0.378
*DDB1*	**2.1**	**0.048**	1.6	0.180	*IL1B*	1.5	0.501	**2.7**	**0.042**
*DNAJA1*	18.7	0.135	**62.1**	**0.0002**	*IL6*	-1.9	0.116	1.0	0.809
*DNAJB4*	8.5	0.180	**22.7**	**0.015**	*MDM2*	15.1	0.108	**33.2**	**0.0009**
*E2F1*	3.7	0.103	**6.7**	**0.0002**	*MT2A*	6.3	0.153	**13.0**	**0.002**
*EGR1*	36.7	0.129	**123.4**	**0.0001**	*NFKB1*	30.0	0.196	**96.6**	**0.004**
*EPHX2*	2.8	0.120	**4.2**	**0.016**	*NFKBIA*	3.9	0.117	**8.5**	**0.015**
*ERCC1*	3.8	0.165	**5.8**	**0.005**	*NOS2*	10.2	0.218	**26.5**	**0.002**
*ERCC3*	1.3	0.371	**1.8**	**0.050**	*PCNA*	1.8	0.346	**4.0**	**0.004**
*FASLG*	9.5	0.172	**32.7**	**0.0005**	*PRDX1*	2.9	0.098	2.4	0.116
*FMO5*	1.4	0.429	**2.7**	**0.015**	*PRDX2*	**3.0**	**0.004**	2.1	0.136
*GDF15*	**4.9**	**0.001**	**3.7**	**0.104**	*PTGS1*	3.2	0.131	**5.6**	**0.0003**
*GPX1*	20.3	0.160	**52.0**	**0.0005**	*RAD50*	4.8	0.210	**14.9**	**0.0004**
*GSR*	**4.6**	**0.055**	**5.4**	**0.015**	*SERPINE1*	3.1	0.212	**8.1**	**0.0016**
*HMOX1*	**2.3**	**0.031**	**1.6**	**0.034**	*SOD1*	1.8	0.322	**3.5**	**0.013**
*HSF1*	**6.8**	**0.054**	**9.2**	**0.002**	*SOD2*	2.7	0.288	**5.8**	**0.001**
*HSPA1A*	**4.2**	**0.008**	**2.4**	**0.008**	*UNG*	4.0	0.127	**5.8**	**0.024**
*HSPA1L*	2.9	0.271	**4.3**	**0.013**	*XRCC1*	9.0	0.223	**31.8**	**0.001**
*HSPA2*	**-2.5**	**0.012**	**-2.3**	**0.035**	*XRCC2*	**2.9**	**0.0009**	**2.8**	**0.046**

The genes were expressed as Fold-change (FC) respect to time (T0). The significant down- and up-regulated genes are highlighted in bold.

To evaluate the effects of anaesthesia methods on interactions between genes, gene ontology analysis of microarray data was performed. According to the gene ontology analysis, genes involved in two principal nodes, such as DNA repair (*ERCC1*, *ERCC3*, *RAD50*, *XRCC1*, *XRCC2*), and stress response (*EGR1*, *HSF1*, *SOD*, *GPX*) were selected and evaluated in all enrolled population by qRT-PCR.

**Fig 6:**
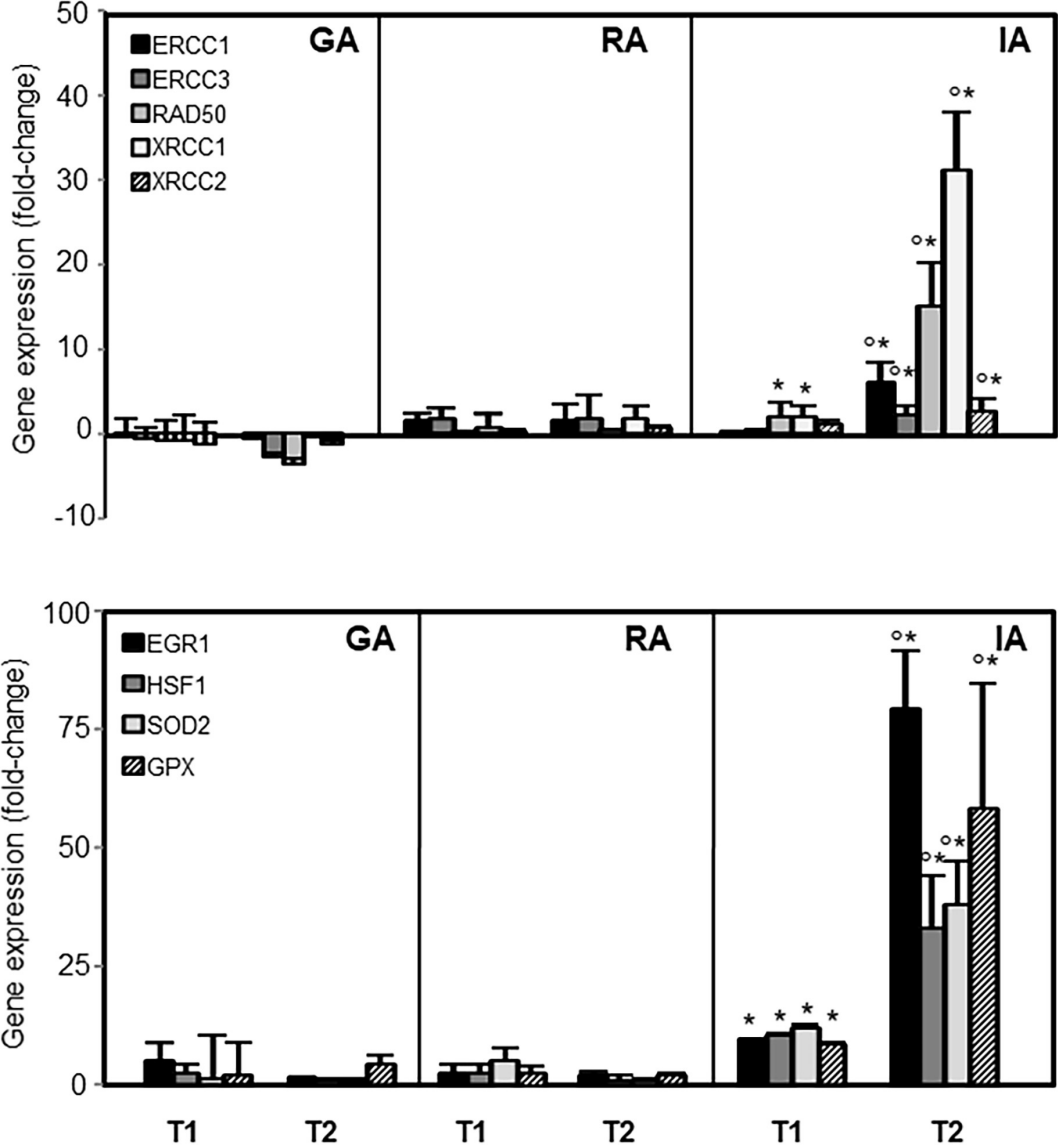
Expression of significantly deregulated stress-responsive genes. Expression of DNA repair proteins (*ERCC1*, *ERCC3*, *RAD50*, *XRCC1*, *XRCC2*), early growth response protein 1 (*EGR1*), heat shock factor 1 (*HSF1*), glutathione peroxidase (*GPX*), and superoxide dismutase (*SOD2*) in patients undergoing arthroplasty before anaesthesia (T0), immediately after operation (T1), and third postoperative day (T2). General anaesthesia (GA, n = 25), regional anaesthesia (RA, n = 25), integrated anaesthesia (IA, n = 25). The data shown are expressed as fold change at T1 and T2 with respect to T0 time points. The symbol ‘*’ denotes significant differences in data at T1 and T2 versus T0 time points, with symbol ‘°’ significance between data at T1 and T2 time points with p<0.05.

As showed in Fig 6, there was agreement between the microarray and qRT-PCR differential expression levels, confirming that IA resulted in a postoperative increase of gene expression. GLM multivariate regression analysis performed to evaluate the association between hepatic and renal marker changes and the anaesthetic method adjusted by age, BMI, gender, and ASA showed that the anaesthesia methods, ASA and sex affected bilirubin changes (Table 5).

**Table 5:** GLM-multivariate regression analyses showing associations between biochemical parameters, anaesthesia and demographic variables.

Model	ΔGOT R = 0.660		ΔGPT R = 0.667		ΔBIL R = 0.385	
	*ήP*	*p*	*ήP*	*p*	*ήP*	*p*
Anaesthesia methods	0.223	0.103	0.239	0.085	0.102	0.381
ASA	0.007	0.726	0.014	0.620	0.001	0.948
Age	0.128	0.121	0.091	0.197	0.038	0.412
BMI	0.011	0.667	0.007	0.725	0.002	0.840
Sex	0.002	0.838	0.013	0.632	0.117	0.139
Anaesthesia methods-ASA	0.038	0.708	0.045	0.658	**0.296**	**0.043**
Anaesthesia methods-Sex	0.077	0.486	0.068	0.528	**0.342**	**0.023**
Anaesthesia methods-ASA-Sex	0.090	0.430	0.102	0.378	**0.314**	**0.17**
ASA-sex	0.095	0.186	0.084	0.214	0.033	0.442
Model	ΔCREA R = 0.589		ΔCPK R = 0.602		ΔHB R = 0.499	
	*ήP*	*p*	*ήP*	*p*	*ήP*	*p*
Anaesthesia methods	0.038	0.709	0.001	0.993	0.065	0.526
ASA	0.007	0.730	0.034	0.434	0.003	0.801
Age	0.028	0.479	0.136	0.110	0.013	0.620
BMI	0.050	0.345	0.016	0.593	0.009	0.680
Sex	0.019	0.567	0.032	0.454	0.001	0.915
Anaesthesia methods-ASA	0.057	0.589	0.007	0.940	0.032	0.732
Anaesthesia methods-Sex	0.116	0.330	0.097	0.400	0.057	0.572
Anaesthesia methods-ASA-Sex	0.039	0.698	0.123	0.306	0.031	0.742
ASA-sex	0.001	0.907	0.001	0.912	0.011	0.649

Partial eta (ήP) indicates the coefficient of correlation and significances (*p*) are highlighted in bold. American Society of Anesthesiologists physical status classification, ASA; Body mass index, BMI; Glutamate Oxaloacetate Transaminase, GOT; Glutamate-Pyruvate Transaminase, GPT; Bilirubin, BIL; Creatinine, CREA; Creatine phosphokinase, CPK; Hemoglobin, HB.

## Discussion

In the present study, we evaluated the influence of three anaesthetic techniques on gene expression in circulating cells at early time point and 3 days after anaesthesia. From the array analysis, we found that among the three anaesthesia methods, RA did not significantly affect gene expression. Deregulated genes were observed in patients under GA, and to a greater extent in subjects undergoing IA, even 3-days after anaesthesia induction, thus implying that multiple molecular processes remain altered over time. The affected genes are known to participate in a wide array of processes including cell cycle and DNA repair, signal transduction, transcriptional regulation, stress response proteins.

Propofol and sevoflurane are widely used in GA, and both have been reported to modulate gene expression [12–14]. Culley et al. first demonstrated that altered gene expression profile in rats induced by GA was associated with persistent changes in hippocampal gene expression, suggesting that recovery of the brain from anaesthesia was considerably slower than generally recognized [15]. Anaesthesia by isoflurane had marked effects on genes in the brain with differential regulation. Gene ontology analysis showed that some genes were functionally related to the anaesthesia and were involved with neurotransmitter release, transport and secretion [16]. We found that most deregulated genes were involved in DNA repair. The gene expression profile revealed an overall down regulation of DNA damage response genes after exposure to propofol, with significant under-expression of genes associated with nucleotide excision repair (*ERCC1*), double strand break repair (*RAD50*, *RAD23A*) and single strand break repair (*XRCC1*). Since up regulation of DNA repair genes occurs in response to DNA damage, a down regulation of DNA repair genes would be expected if a protective effect is induced. The protective effect of propofol was further supported by the down regulation of stress responsive genes, such as the inducible *SOD2* and inflammatory cytokines/chemokines (*TNF*, *IL6*, *CCLs*). The protection of propofol against inflammation and oxidative stress has been previously reported [17–20]. Both propofol and sevoflurane attenuated the extent of hepatic ischemia/reperfusion (I/R) injury by inhibiting Nuclear factor kappa B (NFkB) activation and subsequent alterations in inflammatory cytokines [21,22].

On the other hand, increased DNA damage was observed in PBMCs of subjects who underwent GA: oxidized DNA bases occurred after 15 min of isoflurane exposure associated with an enhancement of DNA repair activity. However, most DNA damage was repaired on the first postoperative day [23]. The mutagen effect of anaesthetics was also observed in occupationally exposed subjects referred as population risk [24]. The isoflurane induced DNA damage as consequence of oxidative stress and inhibition of the repair of DNA damage through the p53 signalling pathway [25]. Besides, desflurane anaesthesia induces DNA strand breaks/alkali-labile sites on the day after minimally invasive surgery in healthy patients [26]. There is controversy over the genotoxic effects of volatile anaesthetics. However, a rise in serum liver enzymes was found in patients who underwent GA, supporting its hepatic toxicity.

No significant alteration in gene expression was found in RA. A toxic effect was observed for local anaesthetics. Lidocaine, bupivacaine, and ropivacaine treatment induced a significant decrease in viability with a concomitant increase in the number of apoptotic cells [27,28]. Mitochondrial DNA damage and decreased ATP and mitochondrial protein levels were found in cells exposed to local anaesthetics [23]. Several mechanisms have been proposed to explain toxicity of local anaesthetics to cells, including the blockade of potassium channels and mitochondrial injury [28]. Rather than single anaesthesia, we reported a synergic interaction between propofol used in GA and regional anaesthetics (IA) activating a set of genes associated with the repair of impaired proteins and structures including genes related to apoptosis, and extensive stress responses probably as result of induced oxidative stress.

Among them, early growth response protein 1 (EGR1) is known as redox-sensitive factor that plays a protective role when cells suffer starvation, ultraviolet light irradiation, hypoxia, and oxidative stress. ROS induce nuclear translocation of apurinic/apyrimidinic endonuclease 1 (APE1), which in turn induces DNA binding of transcriptional regulators such as EGR1 [29]. A number of genes are regulated directly by EGR1, which are highly associated with growth, vascular cell proliferation, cell survival programs and apoptosis [30]. It has been reported that EGR1 binding activity promotes tumour progression or atherosclerosis [31].

Increased expression of the heat shock factor 1 (HSF1) was observed at day 3 of IA induction. HSF1 enable the cell to adapt to various forms of oxidative, electrophilic, thermal, and inflammatory stress by orchestrating elaborate transcriptional programs termed the heat shock response (HSR). The heat-induced genes included many genes known to encode heat shock proteins (HSPs) and other proteins involved in protein folding and degradative pathways [32].

Activation of human heat shock genes was associated by increased antioxidant defence grid that relies on endogenous enzymatic antioxidants (SOD2 and GPX). The induction of the oxidative stress response pathway would suggest a potentially deleterious effect, which affects all forms of major surgery including cardiac surgery, general surgery, trauma surgery, plastic surgery and orthopaedic surgery [33]. This is seen more frequently in arthroplasty, which involves open surgical approaches and, compared to closed surgery, lead to a higher degree of soft tissue injury. The inflammation associated with oxidative stress has been shown to contribute to postoperative complications and to slow down recovery from surgery. In this context, it was reported that patients scheduled for total hip replacement receiving RA showed lower rate of complications and faster circulating cells mass recovery compared with GA and IA [3]. Therefore, anaesthesia procedures may affect postoperative recovery by modulating stress response and RA over the other anaesthesia techniques causes less surgical stress response in terms of changes in stress gene expression. Gene enrichment analysis was performed for regulated genes from microarray; further study using transcriptome sequencing (RNA-seq) technology will quantify overall expression levels and the degree of changes.

## Conclusions

Changes in gene expression in arthroplasty occur early following general anaesthesia, and to a greater extent when integrated with lumbar plexus block, spinal anaesthesia and represents mainly genes involved in stress responses. Surgery itself results in expression of genes whose modulation may prove to be beneficial for acceleration of repair process [34]. Anaesthesia procedure may alter surgery-induced gene expression, thus affecting tissue repair. RA has advantages over the other anaesthesia techniques in terms of changes in stress gene expression.

## Supporting information

S1 File Raw dataset. (PDF)

S2 File Protocol. (DOC)

S3 File CONSORT checklist. (DOCX)

S4 File Study protocol. (XLS)

S1 Table (DOCX)
