# Supplementary material for: Effect of different anaesthetic techniques on gene expression profiles in patients who underwent hip arthroplasty
Source: PLoS One. 2019 Jul 25;14(7):e0219113. doi: 10.1371/journal.pone.0219113 (PMC6657832; doi:10.1371/journal.pone.0219113)
Supplement: S1 File — (PDF) [file pone.0219113.s001.pdf]

S1 File. Protocol

Responsabile della Sperimentazione Prof. Battista Borghi
Responsabile Unità Operativa SSD Coordinamento Ricerca Anestesiologica ove si svolge la sperimentazione Prof. Battista Borghi
Unità Operativa Struttura Complessa Anestesia e Terapia Intensiva Post-operatoria e del Dolore (ATIPD), dott. Stefano Bonarelli
Responsabile Unità Operativa SSD Chirurgia Ortopedica Conservativa e Tecniche Innovative (COTI) ove si svolge la sperimentazione Dr. Dante Dallari
Responsabile Unità Operativa SSD Chirurgia Ricostruttiva Articolare dell'Anca e del Ginocchio ove si svolge la sperimentazione Dr. Ermanno Martucci
Responsabile Unità Operativa SSD Chirurgia di Revisione della Protesi d'anca e Sviluppo Nuovi Impianti (CRA) ove si svolge la sperimentazione Dr. Giovanni Pignatti
Responsabile Unità Operativa Chirurgia Protesica e dei Reimpianti d'anca e di Ginocchio ove si svolge la sperimentazione Dr. Aldo Toni
Unità Operativa SSD Patologia Clinica, dott.ssa Loredana Pratelli
Unità Operativa Struttura Complessa Laboratorio di Oncologia Sperimentale, dott. Piero Picci

1.1 Titolo dello Studio
Effetto degli anestetici usati per l'anestesia regionale vs Generale vs integrata sulla modulazione di geni 'stress-responsivi' coinvolti nel metabolismo e detossificazione cellulare

1.2 Codice identificativo del Protocollo: ____GeSTA________

1.3 Fase della ricerca □ Fase I □ Fase II □ Fase III □Fase IV
(barrare la casella di interesse)

1.4 Studio multicentrico □ SI X NO
Studio internazionale □ SI X NO
(barrare la/e casella/e di interesse)
Se SI N° di centri italiani _____________N° di centri totali _____________
(allegare elenco dei centri partecipanti)
N° pazienti in Italia 99; N° pazienti totali 99; N° pazienti locali; 99
Coordinatore della ricerca _______________Centro di ___________________

1.5.1 Farmaco _____NON APPLICABILE_____ Classe ATC ___________ Ditta _________________
1.5.2 Dispositivo medico ______NON APPLICABILE____________Ditta__________________
1.5.3 Altro (specificare) ____

1.6 In commercio NA □ in Italia □ all'estero □ in quali Paesi

1.7 Indicazioni registrate ________________NA_______________________________

1.8 Sintesi delle premesse teoriche dello studio:
L'inalazione dell'anestetico sevoflurano (SVF) e la somministrazione intravenosa del propofol è una procedura largamente usata nell'anestesia generale. E' stato dimostrato che gli agenti anestetici, tra i quali il SVF, inducono un danno ossidativo al DNA1, ma soprattutto influenzano la funzione epatica nel periodo peri-operatorio. In studi precedenti è stato osservato che gli agenti anestetici modulano l'espressione genica e molti dei geni alterati erano coinvolti nella detossificazione epatica3,4. Sia il SVF che il propofol influenzano il flusso e recupero ematico2, il consumo di ossigeno e la funzione epatica.

Per ovviare i problemi cito-genotossici legati all'anestesia generale, l'anestesia regionale da sola o integrata con l'anestesia generale, costituisce una valida scelta, pertanto è molto usata. L'effetto benefico dell'anestesia regionale è stato osservato in termini di perdite ematiche e recupero ematico postoperatorio2. Sebbene sia ben noto che gli anestetici gassosi usati nell'anestesia generale siano citotossici e che l'uso dell'anestesia regionale riduca lo stress chirurgico, a tutt'oggi non sono stati chiariti i loro effetti tossicologici ed il loro impatto sui sistemi enzimatici di detossificazione.

1.9 Sintesi della letteratura scientifica disponibile (max 5 referenze bibliografiche):

1Alleva R, Tomasetti M, Solenghi MD, Stagni F, Gamberini F, Bassi A, Fornasari PM, Fanelli G, Borghi B. Lymphocyte DNA damage precedes DNA repair or cell death after orthopaedic surgery under general anaesthesia. Mutagenesis. 2003 Sep;18(5):423-8.

2Borghi B, Casati A, Iuorio S, Celleno D, Michael M, Serafini PL, Alleva R. Effect of different anesthesia techniques on red blood cell endogenous recovery in hip arthroplasty. J Clin Anesth. 2005 Mar;17(2):96-101.

3Nakazato K, Yoshida Y, Takemori K, Kobayashi K, Sakamoto A. Expressions of genes encoding drug-metabolizing enzymes are altered after sevoflurane, isoflurane, propofol or dexmedetomidine anesthesia. Biomed Res. 2009 Feb;30(1):17-24.

4Ishikawa M, Tanaka S, Arai M, Genda Y, Sakamoto A. Differences in microRNA changes of healthy rat liver between sevoflurane and propofol anesthesia. Anesthesiology. 2012 Dec;117(6):1245-52.

1.10 Obiettivo dello studio:

Gli obiettivi di questo studio sono:

1. Identificazione di un profilo di espressione genica in relazione alla tecnica anestesiolgica utilizzata.
2. Relazione tra profilo genico e parametri biochimici e clinici di funzionalità epatica, renale e di citotossicità.

1.11 Popolazione dello studio

☐ volontari sani X pazienti

X ricoverati ☐ ambulatoriali ☐ entrambi

1.12 Regime di ricovero dei pazienti nel corso della Sperimentazione:

X ospedaliero ☐ day hospital ☐ day surgery ☐ altro

1.13 Sintesi dei criteri di inclusione:

Verranno considerati eleggibili i pazienti di età compresa tra 18 e 100 anni con ASA I-II sottoposti in elezione ad intervento di artroprotesi d'anca.

Tutti i pazienti dovranno dare il proprio consenso informato scritto per poter essere arruolati e quindi partecipare al protocollo di ricerca.

1.14 Sintesi dei criteri di esclusione:

Pazienti, non collaboranti, di età <18 o >100 anni, con ASA ≥ III, con patologie infiammatorie croniche, neoplastiche, insufficienza epatica e altre patologie con stress ossidativo (malattie reumatologiche e degenerative), pazienti ove sia controindicato l'esecuzione di una delle tre tecniche di anestesia a confronto.

1.15 Disegno dello studio:

☐ non controllato X controllato

☐ vs altro farmaco ☐ vs placebo ☐ vs nessun trattamento

X vs diversa tecnica anestesiologica ☐ vs diversa preparazione farmaceutica

☐ aperto ☐ doppio cieco X singolo cieco ☐ cross-over

X randomizzato non randomizzato
Il cieco riguarda la valutazione del laboratorio e l'analisi statistica:
- dott.ssa Renata Alleva
- dott.ssa Elettra Pignotti

I pazienti saranno randomizzati in tre gruppi sulla base della tipologia di tecnica anestesiologica utilizzata:
1. Anestesia generale
2. Anestesia loco-regionale
3. Anestesia integrata (generale e loco-regionale)

1.16 Trattamenti:

1.17 Eventuali trattamenti concomitanti:
___NA____

1.18 Visite ed esami non di routine previsti dallo studio (vedi allegato 2A):
- Prelievo di sangue intero (10 ml) pre-operatorio
- Prelievo di sangue intero (10 ml) post operatorio (1 giornata)
- Prelievo di sangue intero (10 ml) dopo tre giorni dall'intervento

1.19 Sintesi dei parametri di valutazione dell'efficacia primari e secondari:
- Analisi del profilo di espressione genica attraverso l'RNA isolato dai linfociti: valutazione dell'espressione di 84 geni coinvolti nella tossicità e detossificazione da farmaco, con individuazione della differente espressione in risposta agli anestetici.
- Parametri biochimici di funzionalità epatica (GOT, GPT, Bilirubina totale), funzionalità renale (Creatinina) e di citotossicità (CPK).

1.20 Sintesi dei parametri di valutazione della tollerabilità: NA
1.21 Aspetti farmacoeconomici (qualora sia possibile esplicitare gli elementi di utilità economica derivanti dalla Sperimentazione): NA

1.22 Durata dello Studio (comprensiva di tutte le fasi: reclutamento, trattamento, follow-up) per paziente e in totale:
5 giorni per paziente e 2 anni in totale

1.23 Statistica (esplicitare come è stata calcolata la dimensione del campione):
La numerosità del campione è stata determinata in accordo con Lee- Whitmore. Le assunzioni di base sono che la distribuzione dell'espressione di ciascuno degli 84 geni sia log-normale con deviazione standard su scala logaritmica uguale nei 3 gruppi a pari a 0.7, adatta a geni con moderata- alta espressione e quindi conservativa per la determinazione della numerosità. Desiderando un numero di falsi positivi al massimo pari ad 1, una differenza minima dell'espressione su scala logaritmica tra i gruppi di almeno 0.5 ed una potenza di almeno 0.8 il numero minimo di casi risulta essere di 33 per ciascun gruppo per un totale di 99. L'errore di primo tipo alfa ammonta in tal caso a 0.0119 garantisce un livello di significatività di almeno 0.05 è infatti di sotto del valore di 0.016 richiesto dalla correzione di Bonferroni. La distribuzione normale delle variabili continue sarà valutata mediante test di Kolmogorov-Smirnov. Il test Chi-quadro sarà usato per valutare le variabili categoriche. Le differenze fra i gruppi saranno valutate mediante test parametrici ANOVA seguito da Tukey test. L'analisi di regressione multipla sarà effettuata per valutare l'influenza dei parametri biochimici sull'espressione genica in risposta agli anestetici considerando fattori confondenti quali età, genere, BMI, fumo. Valori di $p < 0.05$ saranno considerati statisticamente significativi. Tutti i test saranno effettuati mediante software (SPSS, Chicago, IL, USA).

1.24 Altre informazioni: NA
1.25 Pianificazione dello Studio:
Data prevista inizio: appena sarà approvato lo studio dal Comitato Etico
Data prevista termine 2 anni dopo la data di inizio

1.26 Proprietà dei dati e uso dei risultati dello studio:
☐ Proprietà esclusiva dello Sponsor
☐ utilizzo dei dati previo assenso scritto dello Sponsor
☐ Consenso congiunto Sponsor e Sperimentatore
X libero

Lo sperimentatore responsabile f.to Battista Borghi

Bologna, 3 aprile 2014
