# Supplementary material for: Effect of different anaesthetic techniques on gene expression profiles in patients who underwent hip arthroplasty
Source: PLoS One. 2019 Jul 25;14(7):e0219113. doi: 10.1371/journal.pone.0219113 (PMC6657832; doi:10.1371/journal.pone.0219113)
Supplement: S3 File — (DOCX) [file pone.0219113.s003.docx]

**S3 File. Study Protocol**

Responsible of the Experimentation: Prof. Battista Borghi

Head of the SSD Operative Unit Coordination of Anesthesiology Research where the trial takes place

Collaboretors:

Dr. Stefano Bonarelli, Anesthesia and Postoperative Intensive Therapy and Pain (ATIPD) Unit.

Dr. Dante Dallari, Head of Operative Unit SSD Orthopedic Conservative Surgery and Innovative Techniques (COTI).

Dr. Ermanno Martucci, Head of Joint Reconstructive Surgery of the Hip and Knee Unit.

Dr. Giovanni Pignatti, Head of Revision Surgery of Hip Prosthesis and Development of New Implants (CRA) Unit.

Aldo Toni, Head of Surgical Surgery Unit and of Hip and Knee Reputations.

Dr. Loredana Pratelli, Operative Unit SSD Clinical Pathology.

Dr. Piero Picci, Operative Unit Complex Structure Laboratory of Experimental Oncology.

**1.1 Title of the Study**

**Effect of anesthetics used for regional vs general anesthesia against the integration of 'stress-responsive' gene modulation for cell metabolism and detoxification**

**1.2 Protocol identification code: ____GeSTA________**

**1.3 Research phase  Phase I  Phase II  Phase III Phase IV**

**(tick the box of interest)**

**1.4 Multi-center study  SI X NO**

**International study  SI X NO**

**(tick the box (s) of interest)**

**If N ° of Italian centers _____________ N ° of total centers _____________**

**(attach list of participating centers)**

**No. of patients in Italy 99; Total number of patients 99; N ° local patients; 99**

**Research coordinator _______________ Center of ___________________**

**1.5.1 Medication _____NON APPLICABILE_____ Class ATC ___________ Company _________________**

**1.5.2 Medical device ______ NOT APPLICABLE____________Ditta__________________**

**1.5.3 Other (specify) ____________________________________________________________**

**1.6 On the market NA  in Italy  abroad  in which countries**

**1.7 Registered indications** [**www.clinicltrials.gov**](http://www.clinicltrials.gov)**, N°** **NCT03585647**

**1.8 Rationale of the study:**

**Inhalation of sevoflurane anesthetic (SVF) and intravenous administration of propofol is widely used in general anesthesia. It has been shown that anesthetic agents, including SVF, induce oxidative damage to DNA (1), affecting hepatic function in the peri-operative period. Previous studies observed that anesthetic agents modulate gene expression and many of the altered genes were involved in hepatic detoxification (3,4). Both SVF and propofol influence blood flow and recovery (2), oxygen consumption and liver function. To overcome cyto-genotoxic effects related to general anesthesia, regional anesthesia alone or integrated with general anesthesia, is a valid choice, therefore it is widely used. The beneficial effect of regional anesthesia was observed in terms of blood loss and postoperative blood recovery (2). Although it is well known that gaseous anesthetics used in general anesthesia are cytotoxic and that the use of regional anesthesia reduces surgical stress, to date the toxicological effects and their impact on the enzymatic detoxification systems have not been clarified.**

**1.9 References (max 5 references):**

**1) Alleva R, Tomasetti M, Solenghi MD, Stagni F, Gamberini F, Bassi A, Fornasari PM, Fanelli G, Borghi B. Lymphocyte DNA damage precedes DNA repair or cell death after orthopedic surgery under general anesthesia. Mutagenesis. 2003 Sep; 18 (5): 423-8.**

**2) Borghi B, Casati A, Iuorio S, Celleno D, Michael M, Serafini PL, Alleva R. Effect of different anesthesia techniques on red blood cell endogenous recovery in hip arthroplasty. J Clin Anesth. 2005 Mar; 17 (2): 96-101.**

**3) Nakazato K, Yoshida Y, Takemori K, Kobayashi K, Sakamoto A. Expressions of genes encoding drug-metabolizing enzymes are altered after sevoflurane, isoflurane, propofol or dexmedetomidine anesthesia. Biomed Res. 2009 Feb; 30 (1): 17-24.**

**4) Ishikawa M, Tanaka S, Arai M, Genda Y, Sakamoto A. Differences in microRNA changes of healthy rat liver between sevoflurane and propofol anesthesia. Anesthesiology. 2012 Dec; 117 (6): 1245-52.**

**1.10 Objective of the study:**

**1. Identification of a gene expression profile in relation to the anesthesiology technique used.**

**2. Relationship between gene profile and biochemical and clinical parameters of hepatic, renal and cytotoxicity.**

**1.11 Population of the study**

** healthy volunteers X patients**

**Inpatient  outpatient  both**

**1.12 Patients admission scheme during the experimentation:**

**X hospital  day hospital  day surgery  other**

**1.13 Summary of inclusion criteria:**

**Patients between the ages of 18 and 80 with ASA I-II undergoing elective hip arthroplasty will be considered eligible.**

**All patients with written informed consent in order to be enrolled and then participate in the research protocol.**

**1.14 Summary of exclusion criteria:**

**Non-collaborating patients, aged <18 or> 100 years, with ASA ≥ III, with chronic inflammatory pathologies, neoplastic, liver failure and other pathologies with oxidative stress (rheumatological and degenerative diseases), patients where it is contraindicated the execution of a of the three anesthesia techniques compared.**

**1.15 Study design:**

** not checked X checked**

** vs other drug  vs placebo  vs no treatment**

**X vs different anesthesiology technique  vs different pharmaceutical preparation**

** open  double blind X single blind  cross-over**

**X randomized  non-randomized**

**The blind man is concerned with laboratory evaluation and statistical analysis:**

**- Dr. Renata Alleva**

**- Dr. Elettra Pignotti**

**1.7 Registered indications ________________NA______________________________**

**Patients will be randomized into three groups based on the type of anesthetic technique used:**

**1. General anestesia**

**2. Anestesia loco-regionale**

**3. Anestesia integrata (generale e loco-regionale)**

**1.16 Treatments:**

**FARMACO DOSE / DIE VIA SOMMIN. N ° SOMMIN. DURATION**

**N / A**

**1.17 Possible concomitant treatments:**

**___N / A_________________________________________________________________________**

**1.18 Visits and examinations not routinely foreseen by the study (see annex 2A):**

**• Withdrawal of whole blood (10 ml) pre-operative**

**• Withdrawal of whole blood (10 ml) post-operative (1 day)**

**• Withdrawal of whole blood (10 ml) three days after surgery**

**1.19 Summary of primary and secondary efficacy evaluation parameters:**

**• Analysis of the expression of 84 genes in drug toxicity and detoxification, with identification of the difference in expression in response to anesthetics.**

**• Biochemical parameters of liver function (GOT, GPT, total Bilirubin), renal function (Creatinine) and cytotoxicity (CPK).**

**1.20 Summary of tolerability evaluation parameters:**

**___________________________________________________________________________________**

**1.21 Pharmacoeconomic aspects (if it is possible to clarify the elements of economic utility deriving from the experimentation):**

**___________________________________________________________________________________**

**1.22 Duration of the study (including all phases: recruitment, treatment, follow-up) per patient and in total:**

**5 days per patient and 2 years in total**

**1.23 Statistics (explain how the sample size was calculated):**

**The sample size was determined according to Lee- Whitmore * and G*Power Ftest for ANOVA Fixed effects, omnibus, one-way (Lee ML, Whitmore GA. Stat Med. 2002;21: 3543-3570). Assuming a Poisson distribution for the expected value of the false-positive gene expression of the 9 chosen genes and fixing at 1 the maximum expected value for false positives E(R_0_) and considering all the 84 genes as not differentially expressed (G_0_=G=9), the probability α for any single gene among the G genes that are not differentially expressed is given by** $\boldsymbol{\alpha}\boldsymbol{=}\frac{\boldsymbol{E}\left( \boldsymbol{R}_{\boldsymbol{0}} \right)}{\boldsymbol{G}}\boldsymbol{=}\frac{\boldsymbol{1}}{\boldsymbol{9}}\boldsymbol{=}\boldsymbol{0}\boldsymbol{.}\boldsymbol{011}$**, with the Bonferroni correction it becomes** $\boldsymbol{\alpha}_{\boldsymbol{c}}\boldsymbol{=}\frac{\boldsymbol{0}\boldsymbol{.}\boldsymbol{011}}{\boldsymbol{3}}\boldsymbol{=}\boldsymbol{0}\boldsymbol{.}\boldsymbol{037}$**. This is the type I error of a false positive expression. Moreover, considering as primary endpoint the fold increase of the gene expression, assuming a log-normal distribution with standard deviation of 0.7 which is typical of moderate-high gene expression and therefore a conservative one for the sample size determination and imposing a minimum difference on logarithmic scale among the 3 groups of 0.5 with a power of at least 0.8 and a Bonferroni corrected type I error α=0.05/3=0.0167 (which is smaller and then conservative, than the previous** $\boldsymbol{\alpha}_{\boldsymbol{c}}$**) the minimum sample size(G*POWER)^ for each group is 30 patients, by considering a 10% of drop-out , the chosen sample size was 33 patients per group which leads to a total sample size of 99 patients.**

^**F tests -** ANOVA: Fixed effects, omnibus, one-way

**Analysis:** A priori: Compute required sample size

**Input:** Effect size f = 0.7

α err prob = 0.0167

Power (1-β err prob) = 0.8

Number of groups = 3

**Output:** Noncentrality parameter λ = 14.7000000

Critical F = 4.7803455

Numerator df = 2

Denominator df = 27

Total sample size = 30

Actual power = 0.8009945

**The normal distribution of continuous variables will be evaluated by Kolmogorov-Smirnov test. The Chi-square test will be used to evaluate categorical variables. The differences between groups will be evaluated by means of parametric ANOVA tests followed by Tukey test. Multiple regression analysis will be performed to evaluate the influence of biochemical parameters on gene expression in response to anesthetics considering confounding factors such as age, gender, BMI, smoking. Values of p <0.05 will be considered statistically significant. All tests will be performed using software (SPSS, Chicago, IL, USA).**

**1.24 Other information:**

________________________________________________________________________________________________________________________________________________________

**1.25 Study Planning:**

**Expected start date: as soon as the study by the Ethics Committee will be approved**

**Expected date 2 years after the start date**

**1.26 Data ownership and use of study results:**

** Exclusive property of the Sponsor**

** use of the data with prior written consent of the Sponsor**

** Joint Sponsor and Investigation Consent**

**X free**

**The experimenter in charge of Battista Borghi**

**Bologna, 3 April 2014**
