# Supplementary material for: Effect of different anaesthetic techniques on gene expression profiles in patients who underwent hip arthroplasty
Source: PLoS One. 2019 Jul 25;14(7):e0219113. doi: 10.1371/journal.pone.0219113 (PMC6657832; doi:10.1371/journal.pone.0219113)
Supplement: S1 Table — (DOCX) [file pone.0219113.s005.docx]

**S1 Table. list of genes used to identify deregulated pathways**
